# The King's National Roll

**Published:** 1920-03-20

**Authors:** 


**THE PUBLIC WELL-BEING**—(*continued*).

### The King's National Roll.

The first edition of the King's National Roll, which is now in circulation, contains nearly 10,000 names of employers who had, up to the end of 1919, given the necessary undertaking to employ an agreed percentage of disabled ex-Service men on their staffs. These employers have in their service, in the aggregate, 1,486,225 workpeople, including 89,619 disabled ex-Service men. Between December 31 and the middle of February the number of names on the roll was increased to 10,867, representing 1,755,431 workpeople, and including 102,011 disabled ex-Service men. These additional names will be included in the next issue of the roll in a few months' time.

The scheme was only launched in September last year, and the progress made may certainly be considered satisfactory. The local employment committees of the Ministry of Labour have left no stone unturned to ensure its success. Dividing the country into districts, they have approached every municipality, local authority, and employer of labour, with the results now stated. That disabled men taken into employment justify the claims made on their behalf is shown by the fact that when employers all over the country were invited to report their experience of the conduct and work of these men, almost all gave gratifying replies. Satisfactory, however, as this first list of firms and employers may be, especially in view of some of the industrial difficulties which have militated against the scheme, there is still much to be done if it is really to serve the purpose for which it was launched. A special canvass is being continued in different districts with a view to arousing the active co-operation of those employers who have not yet given the necessary undertaking. It is hoped as a result of this that the remaining 31,000 disabled men already on the unemployment registers, together with those yet to be received from the hospitals, will soon be found places in industry and be once again earning their livelihood in civilian occupations.

